# Organ-Specific Effects of Low Dose Radiation Exposure: A Comprehensive Review

**DOI:** 10.3389/fgene.2020.566244

**Published:** 2020-10-02

**Authors:** Eunguk Shin, Sungmin Lee, Hyunkoo Kang, Jeongha Kim, Kyeongmin Kim, HyeSook Youn, Young Woo Jin, Songwon Seo, BuHyun Youn

**Affiliations:** ^1^ Department of Integrated Biological Science, Pusan National University, Busan, South Korea; ^2^ Department of Integrative Bioscience and Biotechnology, Sejong University, Seoul, South Korea; ^3^ Laboratory of Low Dose Risk Assessment, National Radiation Emergency Medical Center, Korea Institute of Radiological & Medical Sciences, Seoul, South Korea; ^4^ Department of Biological Sciences, Pusan National University, Busan, South Korea

**Keywords:** low-dose radiation, human, animal model, organ-specificity, biological marker

## Abstract

Ionizing radiation (IR) is a high-energy radiation whose biological effects depend on the irradiation doses. Low-dose radiation (LDR) is delivered during medical diagnoses or by an exposure to radioactive elements and has been linked to the occurrence of chronic diseases, such as leukemia and cardiovascular diseases. Though epidemiological research is indispensable for predicting and dealing with LDR-induced abnormalities in individuals exposed to LDR, little is known about epidemiological markers of LDR exposure. Moreover, difference in the LDR-induced molecular events in each organ has been an obstacle to a thorough investigation of the LDR effects and a validation of the experimental results in *in vivo* models. In this review, we summarized the recent reports on LDR-induced risk of organ-specifically arranged the alterations for a comprehensive understanding of the biological effects of LDR. We suggested that LDR basically caused the accumulation of DNA damages, controlled systemic immune systems, induced oxidative damages on peripheral organs, and even benefited the viability in some organs. Furthermore, we concluded that understanding of organ-specific responses and the biological markers involved in the responses is needed to investigate the precise biological effects of LDR.

## Introduction

Ionizing radiation (IR) is a high energy radiation that can change the status of intracellular nucleotides, proteins, and organic molecules by generating reactive oxygen species (ROS; Kim et al., 2019). Its effect exhibits a dose-dependently increase, and high dose radiation (HDR) has been utilized for eliminating inoperable cancer cells and keloid scars in patients (Rödel et al., 2017). Conversely, low-dose radiation (LDR) exposure occurs during clinical diagnoses – such as X-ray radiography and computed tomography (CT) – continuous nuclear work, or after nuclear accidents (Maqsudur Rashid et al., 2019). Although there are controversies regarding the definition and effects of LDR, many experimental studies consider IR under 0.5 Gy as LDR and have demonstrated that individuals exposed to LDR show significant health risks, including the occurrence of leukemia and cardiovascular diseases (Pearce et al., 2012; Siegel et al., 2017). However, the precise molecular mechanisms activated in response to LDR exposure are still elusive.

As the effects of LDR on humans are subtle and are generally represented as chronic effects, current studies have concluded the effects based on epidemiological studies in human and animal populations exposed to radiation from nuclear power plants (Boice et al., 2019). However, the results of these epidemiological studies are very limited and have identified very few epidemiological markers for further investigations (Braga-Tanaka et al., 2018). Many experimental studies have explored LDR-induced molecular markers in mouse and cell models and emphasized organ-specific sensitivities and responses in the expression patterns of these markers (Lee et al., 2006). Therefore, it is important to comprehend these studies in the context of alterations in biological events and markers in response to LDR exposure from the viewpoint of dose‐ and organ-specificity.

Although little is known about the mechanism of LDR exposure, many studies reported DNA damages, oxidative stress, and pro-inflammatory responses as major mediating events induced by an LDR exposure. Accumulations of the oxidative stresses and persistent pro-inflammatory responses cooperatively alters the cellular and mitochondrial redox balance, forms oxidized nucleotides, and impairs the DNA repair capacity, which results in DNA damages (Dąbrowska and Wiczkowski, 2017; Czarny et al., 2018; Moloney and Cotter, 2018). Previous studies showed that genes in euchromatic regions were more susceptible to both HDR‐ and LDR-induced DNA damage, double-strand break, and r-H2AX accumulation (Puck et al., 2002; Lafon-Hughes et al., 2008; Sak et al., 2015). Therefore, LDR-induced stresses convergently led to the permanent changes in the cells. Therefore, we focused on the risk of biological effects derived from the LDR exposure and suggested the phenotypic marker expressions.

In this review, we summarized the studies on LDR-induced biological effects in humans, which have been supported by various mouse and cell models. We arranged these effects according to dose‐ and organ-specificity, with descriptions based on significantly altered expressions of biological markers and related molecular mechanisms.

## The Effects of LDR in Humans

The harmful effects of LDR were first reported by some epidemiological studies on individuals who were occupationally or accidently exposed to LDR. At that time, the studies warned that LDR could induce DNA damage and its related responses, which highlighted the need for investigations on LDR exposure-induced molecular changes. First, we discussed studies encompassing changes in people exposed to LDR.

A study evaluated the occurrence of mutations in hypoxanthine-phosphoribosyltransferase (HPRT) in workers engaged in the clean-up after the nuclear accident at the Chernobyl nuclear power plant (Thomas et al., 2002). Results showed that LDR (0.09–0.11 Gy) significantly increased deletion mutants in HPRT in peripheral blood cells, and that the mutation frequency declined with time following exposure. This study stressed the importance of early detection of LDR-induced DNA damage, which reduces with the passage of time. In addition, the same study found that the gene expression profile induced by 0.025 Gy was not significantly different from that induced by 0.1 Gy, which suggested a high sensitivity of humans to LDR exposures. LDR also alters gene expression through epigenetic regulation; global methylation levels were lower and chromosome aberrations higher in radiation-exposed workers than that in controls (Lee et al., 2015). Although LDR-induced ROS in humans has not been that well-studied, individuals exposed to LDR (0.004 Gy/year) showed increased H_2_O_2_ production and antioxidant expression, and their cells showed susceptibility to apoptosis (Russo et al., 2012).

Studies on people exposed to LDR after the Chernobyl nuclear power plant disaster showed that LDR > 0.01 Gy increased the expression of cytokine receptors and growth factors in blood monocytes, which suggested LDR induces the inflammatory responses (Albanese et al., 2007). The importance of LDR-induced biological aberrations was also supported by analyses of severe chronic symptoms presented by individuals exposed to the radiation. An epidemiological investigation about the effects of LDR – by European project toward low dose research toward multidisciplinary integration (DoReMi) – revealed that the risk of leukemia and solid tumors increased in response to exposure to LDR < 0.1 Gy, and that the risk of pediatric leukemia, brain tumor, and cardiovascular disease increased after CT scans with a radiation strength of 0.03–0.06 Gy (Pearce et al., 2012; Hall et al., 2017). In addition, the Chernobyl accident victims showed that enhanced CAP-Gly domain-containing linker protein 2 (CLIP2) expression derived from chromosomal mutation in 7q11.23 region (Kaiser et al., 2016). In the context of physiological malfunction, chronic LDR exposure in contaminated region resulted in increased microvessel density through stabilization of hypoxia-inducible factor-1 alpha *via* the activation of Ras/Raf signaling (Romanenko et al., 2012). Hormone levels, including those of thyroxin, cortisol, thromboxane B2, growth hormone, cAMP, cGMP, and 6-ketoprostaglandin F1, were reportedly altered upon LDR exposure in recovery workers working at the site of the Chernobyl accident (liquidators; Souchkevitch and Lyasko, 1997).

Although these studies highlighted the significant involvement of LDR in various symptoms, the underlying molecular mechanisms remain elusive due to the lack of studies about the biological responses and markers of LDR exposure. In the following section, we have listed and summarized the recent experimental studies on LDR exposure for a better understanding of the biological roles of the LDR-specific markers. Although the conditions of irradiation are quite different in each of these mice and cell models, most studies have reported the organ-specificity of LDR-induced biological effects. Therefore, we classified the results based on the tissues of interest with descriptions of the LDR doses used.

## The Organ-Specific Effects of LDR in Cell and Mouse Models

### Peripheral Blood Cells

The alterations in biological markers in blood are widely used for disease diagnosis due to high sample accessibility and variety. Therefore, many studies aimed at identifying the biological markers and studying the effects of LDR exposure used blood specimens. Collective analysis of peripheral blood cells is useful due to its availability. In an analysis of whole white blood cells, exposure to LDR < 0.5 Gy resulted in increased expression of DNA damage-inducible genes (Ishihara et al., 2016). In particular, it was found that the expression of Ku70, Ku80, and H2A histone family member X (H2AX) – but not that of γH2AX – was induced in peripheral blood cells upon prolonged LDR exposure in a rat model (Zhang et al., 2016b). This was supported by another study that demonstrated increased DNA strand breaks, oxidative base damage, and chromosomal aberrations with no changes in the amount of oxidized nucleic bases, following exposure to LDR (0.05 and 0.1 Gy) in human blood cells (Sudprasert et al., 2006). As the expression of DNA repair genes, including that of human 8-oxoguanine DNA N-glycosylase 1 (hOGG1) and X-ray repair cross complementing 1 (XRCC1), was downregulated by LDR (0.05 Gy) – which further decreased in a dose-dependent manner – LDR was suggested to enhance DNA damage and reduce DNA repair capacity in peripheral blood cells. In humoral immunity, LDR exposure decreased white blood cell and platelets counts along with the number of CD3^+^, CD4^+^, and CD8^+^ T cells and CD4^+^/CD8^+^ ratio in peripheral blood cells (Zhang et al., 2016b). In addition, LDR (0.3–0.7 Gy) reportedly reduced leukocyte/endothelial adhesion through inducing the expression of transforming growth factor beta (TGF-β) and interleukin 6 (IL-6), and inhibited leukocyte infiltration into inflammatory site (Roedel et al., 2002). Taken together, LDR could significantly regulate the status of peripheral blood cells by inducing DNA aberrations, suppressing activation, reducing viability, and perhaps inhibiting immunogenicity of peripheral blood cells.

### Immune Cells

Having discussed the effects of LDR on whole peripheral blood cells, we focused on studies performed using sorted immune cells or cell lines. These studies present information regarding cell type-specific molecular events and signaling pathways involved in LDR responses in immune cells. In bone marrow mesenchymal stem cells (BM-MSCs), LDR (0.1 Gy) slowed the expansion of BM-MSCs and induced the differentiation of hematopoietic cells (HPCs) into myeloid progenitor cells (CD34^+^ CD38^+^ cells) through increased expression of IL-6 and decreased expression of FMS-like tyrosine kinase 3 ligand (Flt3L; Novershtern et al., 2011; Fujishiro et al., 2017). In contrast, LDR (0.1–0.2 Gy) promoted the proliferation of BM-MSCs in a dose-dependent manner (Wu et al., 2011). Further, LDR (0.025, 0.075, and 0.1 Gy) enhanced the proliferation and mobilization of HPCs into peripheral blood through increased granulocyte-colony stimulating factor (G-CSF) expression in a mouse model (Li et al., 2004). Although these results could not explain the regulation of proliferation in bone marrow stem cells, it is commonly believed that LDR enhances hematopoiesis through increased secretion of differentiating cytokines.

Innate immunity plays a major role in establishing primary defense mechanisms against antigens *via* inducing the inflammatory response, detecting foreign cells and phagocytosis, and presenting the antigens to adaptive immune cells. Therefore, a change in innate immune cell activity has a direct effect on the regulation of systemic immune responses (Zhang and Mosser, 2008). In microglial cells, LDR < 0.5 Gy was found to reduce oxidative stress by activating superoxide dismutase (SOD) and suppressing the formation of mitochondrial permeability transition pore (mPTP; Betlazar et al., 2016). This is consistent with findings from another study that showed that LDR (0.25 and 0.5 Gy) induced glutathione expression through activation of the activator protein 1 and NF-kB pathway in macrophages (Kawakita et al., 2003). LDR <0.5 Gy reduced the expression and secretion of IL-1β, IL-6, and tumor necrosis factor α (TNF-α) in macrophages (Li et al., 2017). In addition, LDR exposure also resulted in reduced p65 and extracellular signal-regulated kinase (ERK) phosphorylation, suggesting that LDR suppresses the dispersion of macrophages. Several studies have also demonstrated the significant roles of LDR in the regulation of mast cell activation. LDR < 0.5 Gy had no cytotoxic effects on mast cells, but resulted in reduced cytokine expression (TNF-α, IL-4, and IL-13), with subsequent suppression of allergic response (Joo et al., 2015). Consistently, LDR < 0.05 Gy reduced histamine, IL-4, TNF-α, and beta-hexosaminidase expression while LDR < 0.5 Gy suppressed migration and activation of mast cells through downregulation of nuclear receptor subfamily 4 group A member 2 (Nr4A2; Joo et al., 2012; Song et al., 2019). Taken together, studies show that the exposure of innate immune cells to LDR can lead to the suppression of immunity through regulatory imbalance in the levels of inflammatory cytokines and oxidative stress.

On the other hand, investigations regarding the effects of LDR on adaptive immune cells are limited. One study demonstrated that LDR (0.05 Gy) activated T cells by increasing interferon γ (IFN-γ), IL-2 expression, and differentiation rates, even in immune-suppressive environments (Chen et al., 2011, 2014b). Heat shock protein 70 – whose expression is increased by LDR – could aid the delivery of antigenic peptides to T cells *via* indirect antigen presentation pathways (Multhoff et al., 2015). In B cells, LDR (0.1 Gy) reportedly induced expression of miRNAs including let7a, miR-15b, miR-16, miR-21, and miR-23b, which commonly target the lipid biosynthetic enzyme, glycerol-3-phosphate acyltransferase (GPAT; Tabe et al., 2016). As the expression of GPAT correlates with the immunogenic activity of B cells, LDR might help B cell in acquiring immunity. A transcriptome analysis confirmed LDR-induced (0.05 Gy) expression of IFN-γ, IL-4, and IL-6 (Cho et al., 2018). In the same study, LDR also induced the expression of genes involved in mRNA translation, mitochondrial function, cell cycle regulation, and cytokine induction. These studies demonstrated that LDR induced acquisition of immunity of T and B cells through regulation of both intracellular gene expression and cytokine expression. Given that these findings clearly contradict the findings of studies conducted in innate immunity cells, more strictly-controlled investigations are warranted to elucidate the roles of LDR in immunity.

### Skin

The skin is the primary recipient of external radiation, and many studies have investigated molecular alterations in skin models after LDR exposure. In HaCaT cells, LDR (0.1 Gy) induced p21 expression and increased keratinocyte differentiation (Hahn et al., 2016). However, in another study, LDR < 0.1 Gy increased the differentiation of HaCaT cells, but suppressed p21 expression, remaining the disparity of LDR-induced survival changes in HaCaT cells (Son et al., 2019). In a study on the effects of LDR on the survival of HaCaT cells, 0.05 and 0.5 Gy of LDR was found to upregulate the expression of TP53, BAX, BCL-2, and caspases 2 and 6, leading to cell cycle arrest and transduction of pro-apoptotic signals (Furlong et al., 2013). LDR-induced changes were also validated by a metabolomic analysis, which showed that LDR (0.03 and 0.1 Gy) significantly altered the concentration of metabolites involved in DNA/RNA damage and repair and lipid and energy metabolism (Hu et al., 2012). Although the data are few, these studies suggested that LDR could enhance the differentiation of keratinocytes and could modulate cell proliferation through cell cycle regulation and apoptotic gene expression.

In dermal fibroblasts, the expression of collagen type I alpha 1 (COL1A1), matrix metalloproteinase-1, growth/differentiation factor 15, and Connexin 43 was found to be increased by LDR < 0.5 Gy (Glover et al., 2003; Bae et al., 2015; Sándor et al., 2015). Alterations in these molecules indicated that LDR could accelerate the remodeling of the dermal matrix. A transcriptome analysis performed in primary human skin fibroblast compared a list of the most distinctive signaling pathways responsive to LDR and HDR (Ding et al., 2005). This study showed that genes related to the regulation of extracellular matrix (ECM) were induced by both LDR and HDR while genes involved in cytoskeleton and intercellular signaling were responsive only to LDR. Given these results, LDR exposure specifically enhanced gene expression related to ECM remodeling in dermal fibroblasts without significant changes in genomic DNA.

### Liver

The liver is a major organ that controls systemic metabolism and maintains homeostasis in response to external stimuli. Many studies implicated LDR as a problematic factor inducing significant changes in liver function and deregulating some hepatic metabolites involved in homeostasis. Firstly, in a mouse model, LDR (0.25 and 0.5 Gy) increased the hepatic expression and urine level of hepcidin-2 even after 168 h of irradiation (Iizuka et al., 2016). The increase in urine hepcidin levels was related with impairments in iron transport, which was consistent with the findings of another study that showed that X-rays interfered with the maintenance of iron homeostasis in a mouse model (Christiansen et al., 2007).

In addition, many studies have proposed the principal roles for LDR in glucose and lipid metabolism. LDR (0.1 and 0.5 Gy) inhibited the glycolytic pathway and pyruvate dehydrogenase to suppress glucose consumption and inhibited lipid metabolism *via* inactivation of peroxisome proliferator-activated receptor α and got worse liver inflammation (Bakshi et al., 2015). Although it was not clear whether LDR enhances or suppresses lipid oxidation, the alterations in hepatic lipid metabolism in response to LDR exposure were supported by some *in vivo* studies. Accumulative LDR exposure (total 0.02 or 0.4 Gy) significantly decreased the expression of genes involved in non-esterified fatty acid metabolism ontology in mice (Uehara et al., 2010). In mice exposed continuously to LDR, LDR-induced lipid metabolic changes were also found to be influenced by changes in miRNA (miR-21, miR-221, miR-421, miR-155, and miR-375) expression (Liang et al., 2018). In summary, the studies implied that LDR exposure induced liver specific gene alteration leading to imbalanced homeostasis with deregulated iron, glucose, and lipid metabolism in the liver.

### Brain

Although the skull protects the brain from external damages, LDR does penetrate and affect the brain. Experimental studies have provided various evidences about the occurrence of brain damage in response to LDR exposure. A transcriptome analysis showed that LDR (0.1 Gy) mediated gene expression change related to DNA damage response, ion channels regulation, long-term potentiation/depression, and vascular damage in the brain tissues of mice (Lowe et al., 2009). Furthermore, LDR-induced (0.1 Gy) DNA damage was validated by increased expression of genes involved in DNA repair [protein tyrosine phosphatase non-receptor type 1 (PTPN1), PMS2, high mobility group nucleosomal binding domain 2 (HMGN2), and interferon regulatory factor 3 (IRF3); Birger et al., 2003; Furusawa and Cherukuri, 2010; Yu et al., 2015; Mojena et al., 2018]. These aberrant changes induced by LDR also led to the activation of survival signaling pathway in the brain. LDR < 0.5 Gy induced the expression of cytokine IL-8, leading to the activation of NF-κB and upregulation of NF-κB target genes [intercellular adhesion molecule 1 (ICAM-1), vascular cell adhesion molecule 1 (VCAM-1), and cyclooxygenase-2 (COX2)] in the brain (Manna and Ramesh, 2005; Veeraraghavan et al., 2011). In addition, NF-κB activated by LDR (0.01 and 0.5 Gy) reportedly increased SOD2 expression, which triggered a NF-κB-SOD2-NF-κB positive feedback cycle (Veeraraghavan et al., 2011; Bevelacqua and Mortazavi, 2018). Studies also reported that the damages induced by LDR resulted in neuronal defects. In a mouse model, LDR (0.075 Gy) decreased cAMP levels and cAMP/cGMP ratio in the neurons of the hypothalamus, which was related to the suppression of neurite growth and regeneration (Wan et al., 2001; Batty et al., 2017). An analysis of mice exposed to LDR (0.05 Gy) revealed that the expression of pre‐ and post-synaptic markers (Synapsin 1, Synaptophysin, Synapse-associated protein 97, Debrin 1, and Postsynaptic density protein 95) was significantly increased, which may cause impairments in cognition and learning (Howe et al., 2019). This is consistent with the findings of a study that showed that LDR (0.5 Gy) had a profound effect on the brain, as evidenced by precursor cell dysfunction and defects in cognition (Silasi et al., 2004). In summary, exposure of brain tissues to LDR can result in detrimental biological marker alterations mediated by oxidative stress and DNA damage, and possible impairment of learning and memory.

### Heart

Atherosclerosis is one of the most concerning disease found in those exposed to IR (Barjaktarovic et al., 2013a). Although a precise molecular mechanism about this phenomenon has not been suggested yet, we summarized previous studies covering the effects of LDR on the heart tissues to find a common conclusion. Some studies insisted that LDR induced harmful effects on cardiac tissue by showing that LDR (0.04 Gy) significantly increased oxidative stress and downstream responsive protein expressions in cardiac tissues (Barjaktarovic et al., 2013a; Horot and Tkachenko, 2017; Seawright et al., 2017). Furthermore, LDR-induced oxidative stress was led to non-transient alteration in metabolism of cardiac muscles and malfunctioning (Barjaktarovic et al., 2013b). In the aspect of metabolic alteration, two studies commonly reported LDR suppressed lipid metabolism with evidence that LDR (0.28 Gy) increased cardiac expression of miRNAs leading to lipid metabolism suppression and chronic internal/external LDR exposure (0.1 mGy/day) showed reduced lipid catabolism and mitochondrial oxidation (Orekhova and Modorov, 2017; Liang et al., 2018). These oxidative stresses possibly mediated heart specific biological effects according to a study that LDR (0.2 Gy) induced DNA damage and its responsive genes expression in primary human fibroblast cell from heart (Grudzenski et al., 2010). However, completely opposite results were suggested in studies to investigate the molecular events after LDR exposure in atherosclerosis models. LDR under 0.1 Gy was delivered to several cardiovascular disease models induced by type 1 diabetes, Apolipoprotein E deficiency, and doxorubicin treatment and reduced oxidative stress and inflammation in the heart were found in the models (Zhang et al., 2011, 2016a; Mathias et al., 2015; Jiang et al., 2017). Therefore, LDR may be a threat for the cardiac tissue impairment, as well as relieve the cardiac damages through regulation of oxidative stress and pro-inflammatory responses.

### Thyroid

The thyroid is one of the most sensitive organs against to LDR exposure from CT, occupational radiation exposures, or nuclear accidents (Sinnott et al., 2010). Most of the studies on the thyroid have assessed the effect of exposure to radiation originating from CT, while a few studies reported on the biological events induced by experimental LDR. LDR < 0.5 Gy could induce increase the significant risk of mediated by ROS generation and DNA damage-responsive expressions of γH2AX and p-p53 (Ser15) in the thyroid (Kanagaraj et al., 2015; Kurashige et al., 2017; Visweswaran et al., 2019).

These damages were validated by histological changes (increase in thyroid gland follicle size, nucleic size, colloid density, and induction of hemorrhage) and functional changes (reduced thyroid hormone synthesis and secretion) in mouse models (Nadol’nik et al., 2003, 2005; Pavlov et al., 2013). LDR-induced thyroid dysfunctions were also supported by the studies, demonstrating unique effects of LDR in autoimmune thyroiditis mouse models. In these studies, LDR (0.5 Gy) consistently promoted the development of thyroiditis through increased autoimmunity; these findings are similar those observed in patients exposed to radiation (Nagayama et al., 2008, 2009). Conversely, LDR < 0.1 Gy protected the thyroid from mutation-induced carcinogenesis (Kaushik et al., 2019). Taken together, LDR can easily damage the thyroid through generation of the significant DNA damages, which are determined by the total dose of radiation.

### Lung

Many studies have shown that the lung ranks among the organs that are most resistant to LDR damage. LDR (0.05–0.6 Gy) did not induce significant biological damage in the lung (Sypin et al., 2003; Kim et al., 2007; Jangiam et al., 2018; Puukila et al., 2019). Although many studies opposed it through showing LDR (0.1 and 0.2 Gy) reportedly promoted cell death, inflammation, ROS generation (lipid oxidation), and DNA damage (5-hydroxymethylcytosine); and activated damage responses (p53, p38, p21, ERK1/2, NF-κB, TGF-β, etc.) in the lung, these studies unanimously agreed that certain irradiation conditions resulted in less severe changes in lung than that in the liver and the spleen (Sypin et al., 2003; Avti et al., 2005; Lee et al., 2006; Kim et al., 2007, 2015; Hong et al., 2014; Jangiam et al., 2018). Moreover, the protective roles of LDR in the lung have been demonstrated in various stress models. In these studies, LDR up to 0.5 Gy suppressed ROS generation, attenuated inflammatory responses (IL-1β, IL-5, IL-6, and TNF-α), delayed senescence, and reduced adverse effects induced by HDR (Kim et al., 2007, 2015; Hong et al., 2014; Liang et al., 2016; Velegzhaninov et al., 2018; Park et al., 2019; Son et al., 2019). Given these results, the lung may be more resistant to LDR exposure than other organs and seems to have a high threshold for LDR-induced lung specific biological marker alteration. Although the precise reason of pulmonary resistance against LDR, it is supposed to be based on the lung-specific antioxidant gene expression to modulate oxidative stress (Kim et al., 2018).

### Spleen

The spleen consists of large number of pulps where peripheral blood cells get collected, activated, or removed; and molecular crosstalk between the spleen and peripheral blood cells determines the systemic immune responses. Similar to the reports on LDR-regulated oxidative stress and inflammatory signaling in peripheral blood cells, studies on the spleen also reported LDR-mediated control of tissue damage. In mouse models, LDR (0.02 and 0.2 Gy) protected the tissue from oxidative stress by increasing the synthesis and recycling of GSH *via* the expression of glutamate-cysteine ligase modifier subunit (GCLM), GSH synthase, and glutathione peroxidase (Lee et al., 2013). This antioxidant effect of LDR was validated in other studies where LDR < 0.1 Gy reduced following HDR-induced damages (Yoshida et al., 1993; Bannister et al., 2015). Another investigation on the effect of LDR on splenocytes demonstrated that LDR (0.02 Gy) did not induce significant damage in cells, and instead slowed the rate of clearance and turnover of damaged cells (Bannister et al., 2015). As pretreatment with LDR (0.1 Gy) even attenuated the stress-induced inflammation of the spleen, the protective roles of LDR in the spleen may be evident (Bannister et al., 2015; Son et al., 2019). However, other studies reported that LDR > 0.1 Gy significantly induced apoptosis in the spleen *via* inducing the expression of p53 and p21 (Lee et al., 2006; Bannister et al., 2015). A few reports on the relationship between the spleen and immune cells also demonstrated the protective effects of LDR. LDR < 0.1 Gy increased the percentage of the CD4^+^ T cell subpopulation while dendritic cell and macrophage counts were reduced in response to short-term exposures (Song et al., 2015). However, long term LDR exposure reduced the percentage of the CD8^+^ or CD28^+^ T cell subpopulation. Interestingly, the common findings from these studies demonstrated a high sensitivity of the spleen with respect to LDR-induced effects that are not similar to those found in other tissues, such as brain, liver, lung, and testis. These results suggest that the LDR induced both harmful and beneficial biological effects in the spleen and followed a hormesis model of LDR-induced responses with a threshold of 0.1 Gy.

### Endothelium

The endothelium refers to cells that line the inner surface of blood vessels and lymphatic vessels. Endothelial cells are composed of simple, single-layered, squamous cells that have direct contact with the blood and lymph. As blood cells are closely associated with endothelial tissues, the changes in the endothelium in response to LDR exposure have been discussed here. According to the studies, LDR was found to make human umbilical vein endothelial cells (HUVECs) more sensitive to pro-inflammatory activation. LDR < 0.5 Gy could activate NF-κB signaling pathway through both increased expression and enhanced transcriptional activity (Prasad et al., 1994; Rödel et al., 2004; Cervelli et al., 2014). LDR (<0.5 Gy) promoted the formation of pro-inflammatory environments by an increase in epithelial ICAM-1 expression and an activation of the adhesion and migration of leukocytes towards inflammatory sites (Voisard et al., 2007; Cervelli et al., 2014). In addition, LDR induced the expression of pro-inflammatory cytokines (IL-8, G-CSF, and platelet-derived growth factor-BB) in epithelial cells, which resulted in a microenvironment that is highly susceptible to inflammation (Meeren et al., 1997; Schröder et al., 2019). LDR (0.1 Gy) increased the expression of endothelin 1, a pro-inflammatory and fibrotic inducer, in HUVECs (Lanza et al., 2007). Further, LDR (0.05 and 0.072 Gy) was found to induce proliferation and migration through ERK signaling in endothelial progenitor cells; this result serves as an evidence for LDR-induced endothelial inflammation (Wang et al., 2019). With the pro-inflammatory responses, LDR (<0.5 Gy) could induce double-strand breaks of DNA and activate DNA damage responses in endothelial cells supported by a microarray data (Mouw et al., 2017). Taken together, LDR is implicated as a major inductor of cellular and environmental inflammation response and DNA damages in the endothelial tissue.

### Osteoblast/Clast

Regulation of the balance between osteoblasts and osteoclasts is important for bone growth and maintenance. The best microstructure for bone growth is formed through repeated formation and absorption. In adults, highly activated osteoclasts in bone tissue can induce arthritis and osteoporosis through bone absorption and inflammation (Lee et al., 2017). Studies that investigate the effects of LDR on osteoblasts and osteoclasts have assessed the alterations in differentiation, proliferation, and activity. Irradiation with LDR (<0.5 Gy) resulted in enhanced differentiation of osteoblasts (Chen et al., 2014a; Lima et al., 2017; Yang et al., 2017). It was evidenced by the activation of Wnt and NF-κB signaling transduction and the expression of differentiation markers [COL1A, alkaline phosphatase, osteocalcin (OCN), core-binding factor alpha 1, and osteoprotegerin; Xu et al., 2012; Chen et al., 2016, 2017; She et al., 2016; Deloch et al., 2018a]. In addition, LDR < 0.5 Gy was found to increase the proliferation of BM-MSCs and osteoblasts, which led to activation of osteoblasts (Chen et al., 2014a; Yang et al., 2017). With respect to osteoclast, multiple studies have reported that LDR (0.5 Gy) suppresses the differentiation into osteoclasts and bone resorbing activities, which led to enhanced bone formation and ameliorated arthritis (Deloch et al., 2018a,b). From these studies, it seems that LDR protects and promotes bone growth by regulating genes related to supporting the proliferation of osteoblast/osteoclasts.

### Testis and Ovary

The testis is a reproductive organ which must be protected from mutations. Testicular tissues are highly vulnerable to damages induced by LDR exposure. LDR < 0.2 Gy increased the levels of ROS, which leads to induction of ER stress and apoptosis in testicular cells of irradiated mice (Wang et al., 2013). Long-term LDR exposure also altered the expression of miRNAs to induce apoptosis through oxidative stress in mouse testes (Liang et al., 2018). However, in the state of inflammation – in the background of type 1 diabetes – LDR < 0.05 Gy has been reported to attenuate apoptosis in mouse testicular tissues (Zhao et al., 2010). Given these results, LDR may pose a major threat to testicular tissue due to aberrant gene expression, despite its protective effects with respect to hyperinflammation.

Only few studies have assessed the changes induced by LDR exposure in other reproductive organs, although the changes reported were significant. LDR (0.05 Gy) protected oocytes from HDR exposure-induced DNA damage (Jacquet et al., 2008). Conversely, LDR (0.1 Gy) significantly reduced the number of follicles as both short‐ and long-term effects (Kimler et al., 2018). In addition, LDR (0.36 Gy) reduced 50% of the germ cells in ovaries of prenatal and neonatal mice (Rönnbäck, 1983). Taken together, LDR also showed hormetic effects in the ovary, while the effective threshold was further lower than that in other organs.

## Conclusion

**Figure 1:**
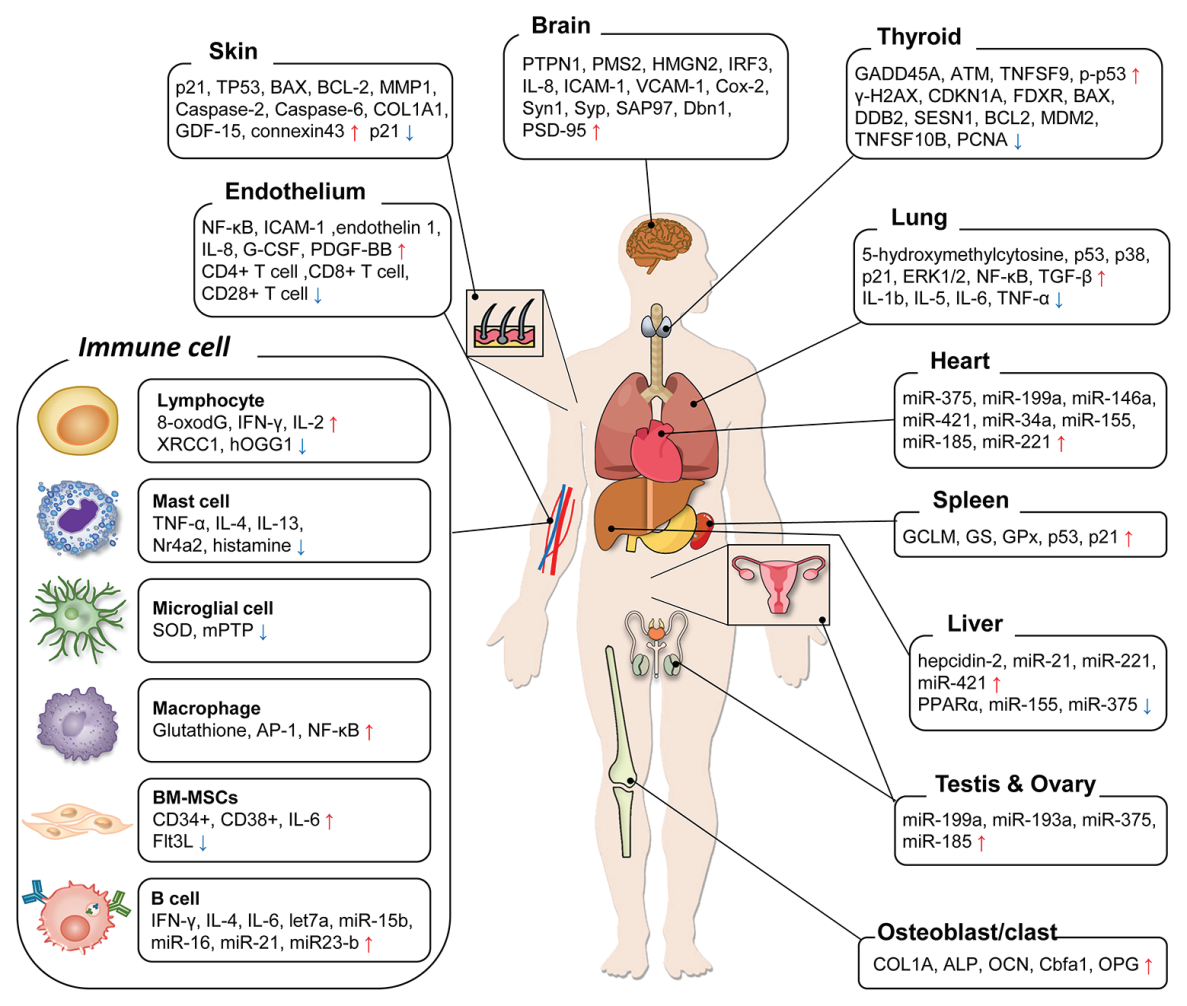
A diagram summarizing organ-specific biological markers by low-dose radiation (LDR) exposure. The molecular markers suggested in previous LDR studies were categorized. For each organ, the markers were enumerated following the expression change after LDR exposure and increase/decrease was marked as arrows (↑/↓).

Determination of LDR exposure is an important problem for continuity of nuclear works and epidemiological analysis of nuclear accidents. However, the effects of LDR are subtle, and the absence of reliable biological markers has been obstacles. In this review, we summarized the biological markers caused by LDR in human, mouse, and cell studies for better understanding of the effects of LDR. Due to the limited studies that have investigated the changes in humans, most of the studies reviewed here were based on the findings in experimental models. The alterations of biological markers by LDR were summarized in Figure 1 and their related molecular events were listed with corresponding references in Table 1. By suggesting promising molecular markers expressed in each organ, we look forward to further studies to discover the LDR specific molecular makers based on this review.

**Table 1:** Organ-specific molecular events and the markers regulated by LDR exposure.

Molecular events	Organ	Molecular marker	LDR dose (Gy)	Reference
DNA damage response	Liver	miR-21, miR-221, miR-421, miR-155, miR-375	Chronic LDR	Liang et al., 2018
	Brain	PTPN1, PMS2, HMGN2, IRF3	0.1	Birger et al., 2003; Furusawa and Cherukuri, 2010; Yu et al., 2015; Mojena et al., 2018
	Heart	miR-375, miR-199a, miR-146a, miR-421, miR-34a, miR-155, miR-185, miR-221	Chronic LDR	Liang et al., 2018
	Thyroid	GADD45A, ATM, TNFSF9, p-p53, γ-H2AX, CDKN1A, FDXR, BAX, DDB2, SESN1, BCL2, MDM2, TNFSF10B, PCNA	4.7–242.5	Visweswaran et al., 2019
	Lung	p53, p38, p21, ERK1/2, NF-κB, TGF-β	0.1, 0.2	Sypin et al., 2003; Avti et al., 2005; Lee et al., 2006; Kim et al., 2007, 2015; Hong et al., 2014; Jangiam et al., 2018
	Peripheral blood cell	Ku70, Ku80, H2AX	Chronic LDR	Zhang et al., 2016b
		8-oxodG	0.05, 0.1	Sudprasert et al., 2006
		XRCC1, Hogg1	0.05	
Immune response	Liver	PPARα	0.02	Bakshi et al., 2015
	Brain	IL-8, ICAM-1, VCAM-1, Cox-2	0.5	Manna and Ramesh, 2005; Veeraraghavan et al., 2011
	Lung	IL-1β, IL-5, IL-6, TNF-α	0.5	Kim et al., 2007, 2015; Hong et al., 2014; Liang et al., 2016; Velegzhaninov et al., 2018; Park et al., 2019; Son et al., 2019
	Endothelia	NF-κB, ICAM-1, ET-1, IL-8, G-CSF, PDGF-BB	<0.5, 0.01, 0.1	Prasad et al., 1994; Meeren et al., 1997; Rödel et al., 2004; Lanza et al., 2007; Voisard et al., 2007; Cervelli et al., 2014; Schröder et al., 2019
	Mast cell	TNF-α, IL-4, IL-13, Nr4a2	<0.5	Joo et al., 2015
		Histamine, IL-4, TNF-α, β-HEX A	<0.05	Joo et al., 2012; Song et al., 2019
	BM-MSC	CD34, CD38, IL-6, Flt3L	0.1	Fujishiro et al., 2017
	Peripheral blood cell	IFN-γ, IL-2	0.05	Chen et al., 2011, 2014b
	Macro-phage	IL-1β, IL-6, TNF-α	<0.5	Li et al., 2017
	B cell	Let7a, miR-15b, miR-16, miR-21, miR-23b, IFN-γ, IL-4, IL-6	0.05, 0.1	Tabe et al., 2016; Cho et al., 2018
Oxidative stress response	Lung	IL-1β, IL-5, IL-6, TNF-α	0.5	Kim et al., 2007, 2015; Hong et al., 2014; Liang et al., 2016; Velegzhaninov et al., 2018; Son et al., 2019
	Spleen	GSH, GCLM, GS, GPx	0.02, 0.2	Lee et al., 2013
	Testis	miR-199a, miR-193a, miR-375, miR-185	Chronic LDR	Liang et al., 2018
	Macro-phage	SOD, GSH, AP-1, NF-κB	0.25, 0.5	Kawakita et al., 2003; Betlazar et al., 2016
Cell proliferation	Skin	p21, TP53, BAX, BCL-2, Caspase-2, Caspase-6	0.05, 0.1, 0.5	Furlong et al., 2013; Hahn et al., 2016; Son et al., 2019
	Osteoblast/clast	COL1A, ALP, OCN, Cbfa1, OPG	Chronic LDR	Xu et al., 2012; Chen et al., 2016, 2017; She et al., 2016; Deloch et al., 2018a
ECM remodeling	Skin	COL1A1, MMP1, GDF-15, Connexin43	<0.5	Glover et al., 2003; Bae et al., 2015; Sándor et al., 2015
Iron metabolism	Liver	Hepcidin-2	0.25, 0.5	Iizuka et al., 2016
Synapto-genesis	Brain	Syn1, Syp, SAP97, Dbn1, PSD-95	0.05	Howe et al., 2019

Although previous studies suggested that there are quite a few differences of the marker expressions among organs by LDR exposure, the molecular basis about the organ-specific sensitivity against LDR exposure has not been well-studied. We observed that LDR generally suppressed innate immune system, while established pro-inflammatory environments for adaptive immune cells. In peripheral organs and brain, LDR commonly induced DNA damages and oxidative stresses, which led to systemic aberrations. Oppositely, hormetic effects of LDR were barely shown in studies about spleen, osteoblasts, and ovary. In a recent study, the concentration of radionuclides was measured in several organs of 79 cattle around the Fukushima Daiichi nuclear power plant (Fukuda et al., 2013), and the deposition of radionuclides was different depending on the organs examined. The radionuclides deposition in muscles was top ranked among the organs due to proximity to skin, but brain, which is covered by skull, showed lower deposition of radionuclides compared to most other organs. This result suggested that even with whole-body irradiation, the irradiation dose and the effect of LDR might vary depending on the location of the organ. Collectively, a convoluted understanding of the organ-specific LDR responses and sensitivity against LDR can be a promising strategy to figure out the core molecular markers.

Although many epidemiological studies about radiation accident or LDR exposure have suggested the risk ratio of diseases, including cancers and cardiovascular diseases, they only could utilize the irradiation doses as a risk factor (Kreuzer et al., 2015; Shore et al., 2017; Hauptmann et al., 2020). As the measuring was performed in various sites (colon, stomach, skin, etc.), it is difficult to interpret the epidemiological analysis precisely. In recent epidemiology studies, the introduction of associated biological markers not only enhanced the accuracy of the analysis but also enabled early diagnosis of the diseases (Sjaarda et al., 2018; Elliott et al., 2019). Furthermore, in the studies covering a stimulus and disease with organ-specific cytotoxicity, the significance of measuring organ-specific biomarkers greatly increases (Tajima et al., 2019; Lieske et al., 2020). Therefore, further epidemiological LDR research is warranted to consider the utilization of biological markers for its risk estimation and pro/retrospective analysis. After all, we hope this review can be relayed to the next generation as an inspiration for further research to find out LDR-specific molecular markers based on biological basis.

## Author Contributions

ES, SL, HK, and BY: conceptualization. ES, SL, HK, and BY: writing original draft preparation. ES, SL, HK, JK, KK, HY, YJ, SS, and BY: writing review and editing. BY: supervision and project administration. All authors contributed to the article and approved the submitted version.

### Conflict of Interest

The authors declare that the research was conducted in the absence of any commercial or financial relationships that could be construed as a potential conflict of interest.

## Abbreviations

BM-MSC: Bone marrow mesenchymal stem cell;
COL1A1: Collagen type I alpha 1;
CT: Computed tomography;
ECM: Extracellular matrix;
ERK: Extracellular signal-regulated kinases;
G-CSF: Granulocyte-colony stimulating factor;
H2AX: H2A histone family member X;
HDR: High dose radiation;
HIF-1α: Hypoxia-inducible factor-1 alpha;
HPC: Hematopoietic cell;
HPRT: Hypoxanthine-phosphoribosyltransferase;
HUVECs: Human umbilical vein endothelial cells;
IFN: Interferon;
IL: Interleukin;
IR: Ionizing radiation;
LDR: Low dose radiation;
NF-kB: Nuclear factor kappa-light-chain-enhancer of activated B cells;
ROS: Reactive oxygen species;
SOD: Superoxide dismutase;
TGF-β: Transforming growth factor beta.
